# Elevating anthocyanin extraction from mangosteen pericarp: A comparative exploration of conventional and emerging non-thermal technology

**DOI:** 10.1016/j.fochx.2024.101882

**Published:** 2024-10-06

**Authors:** Giroon Ijod, Nur Izzati Mohamed Nawawi, Rabiha Sulaiman, Mohammad Rashedi Ismail-Fitry, Noranizan Mohd Adzahan, Farooq Anwar, Ezzat Mohamad Azman

**Affiliations:** a Department of Food Technology, Faculty of Food Science and Technology, 43400, Universiti Putra Malaysia, Selangor, Malaysia; b Department of Food Science, Faculty of Food Science and Technology, 43400, Universiti Putra Malaysia, Selangor, Malaysia; c Institute of Chemistry, University of Sargodha, Sargodha 40100, Pakistan

**Keywords:** Browning enzyme, Colorant, Cyanidin-3-*O-*glucoside, Cyanidin-3-*O*-sophoroside, High pressure processing, Natural pigment, Scanning electron microscope

## Abstract

Mangosteen pericarp (MP) is abundant in bioactive compounds but is often discarded as waste, leading to environmental pollution. This study compared the extraction of dried MP using maceration and high-pressure processing (HPP). HPP at 10 min (500 MPa/20 °C) resulted in the highest ACNs, total phenolic content (TPC), total flavonoid content (TFC), and antioxidant activities. It also significantly reduced residual enzyme activities (REA) of polyphenol oxidase (PPO) and peroxidase (POD) by 33.90 % and 8.27 %, respectively. Fourier-transform infrared spectroscopy (FT-IR) analysis revealed a new wavelength at 2665.25 cm^−1^, and scanning electron microscopy (SEM) showed significant pore formation in MP cells, indicating cell damage. HPP-10 min enhanced the extraction of bioactive compounds, which significantly elevated the thermal stability of ACNs at 60 °C. This suggests that HPP is a promising method for extracting and preserving ACNs and other bioactive compounds from dried MP, with potential applications as natural colorants.

## Highlights

• High pressure processing enhanced anthocyanins and bioactive compound extraction
• High pressure processing reduced activities of PPO and POD enzymes
• High pressure processing improves anthocyanin stability at 60 °C over maceration
• Pores formed in MP cell walls after high pressure processing extraction

## 1 Introduction

Mangosteen (*Garcinia mangostana* L.) is a tropical fruit Native to Southeast Asia. It has been recognized as a queen of fruit because of its unique taste and soft and tangy flavor (Kaur, Singh, & Dar, 2020). The production of mangosteen was 5.9 million tons in 2022 (FAOSTAT, 2023); however, the flesh of mangosteen comprises only approximately 30 % of the whole fruit, resulting in approximately 4.13 million tons of by-product. Numerous bioactive compounds in MP, such as xanthones (α-, β-, and γ-mangostin), flavonoids (anthocyanins (ACNs)), and tannins, have been widely studied for their health benefits (Do & Cho, 2020; Nawawi et al., 2023a). This can be related to anti-obesity, anti-inflammatory, improved gut microbiota, anti-neuroinflammatory and anti-microbial (Albuquerque et al., 2023; Do & Cho, 2020; Kaur et al., 2020).

ACNs are natural pigments in plants that are responsible for the vibrant purple color of MP (Deylami, Rahman, Tan, Bakar, & Olusegun, 2016; Nawawi et al., 2023a). This compound has gained considerable interest due to its strong antioxidant properties and potential use as a natural colorant in various industries, such as food, pharmaceuticals, and cosmetics (Ijod et al., 2024; Netravati et al., 2024). ACNs are a promising alternative to synthetic colorants, especially as consumer demand for natural and health-conscious ingredients continues to grow (Netravati et al., 2024). However, extracting ACNs from plant materials using the conventional method, such as maceration, can be challenging and time-consuming (Ijod et al., 2022). During maceration, mass transfer issues occur due to incomplete extraction, as some compounds bind to the cell membranes of the plant matrix (Netravati et al., 2024). Therefore, HPP has become a preferred alternative for extracting bioactive compounds.

Over the years, the adoption of HPP in the industry become more significant. According to Houška et al. (2022), nearly 600 units of HPP were installed in the production sector. The use of HPP covered in the preservation of juice and beverages, fruits, fruit puree, raw coconut milk, and paste (Houška et al., 2022; Nawawi et al., 2023b; Nowacka et al., 2021), The action of the HPP is based on two basic principles: the Pascal principle and the isostatic principle. Based on the isostatic principle, in a closed environment, any point on the targeted product experiences the same pressure promptly and equally. Meanwhile, the Pascal principle, explained by Le Chatelier's principle, states that when an external force is applied to a stationary fluid in a sealed container, pressure changes are evenly and efficiently transmitted to each point inside the fluid and container walls. This phenomenon ensures that the pressure variations are distributed uniformly and without loss (Nawawi et al., 2023b; Nowacka et al., 2021). HPP is a non-thermal food preservation and extraction method that uses high pressure (typically between 300 and 600 MPa) (Nawawi et al., 2023b; Nowacka et al., 2021). During extraction, the high pressure causes mechanical disruption of plant cell walls and membranes.

The uniform pressure applied in HPP causes the main component of the cell wall (pectin, cellulose and hemicellulose) to change their integrity and binding properties (Hilz, Lille, Poutanen, Schols, & Voragen, 2006). Due to the pressure-sensitive of the non-covalent interaction (hydrogen bonds, ionic bonds, ester bonds, and Van der Waals forces) between the cell walls or membranes, therefore, the breaking and dissociation of the linkages occur, affecting the structure of the cells. This disruption increases cell permeability and can cause the cell walls to rupture, which releases bioactive compounds such as ACNs, flavonoids, and other phytochemicals into the surrounding medium. The increased permeability of the plant cells allows for better penetration of any solvent used during extraction, which enhances the mass transfer of bioactive compounds from the plant matrix into the solvent. High pressure can cause the denaturation of proteins and inactivation of enzymes. This is beneficial in preventing enzymatic degradation of sensitive bioactive compounds during extraction (Nawawi et al., 2023b; Nowacka et al., 2021).

Over the years, increasing the population and changes in diet patterns, demanding healthier diets has caused an increase in fruit and vegetable production worldwide, eventually leading to substantial mass losses, ranging from seeds, pericarps, skins, and pomace, which represent 10–35 % of the fresh material and contribute to greenhouse gas emissions (Ayele, Masi, Ebrahim, & Gamachis, 2024). However, these by-products are rich in phytochemicals and antioxidants, which can be extracted and utilized as value-added ingredients in food products. Traditional thermal extraction methods often result in the degradation of heat-sensitive bioactive compounds found in plant matrices (Yuan et al., 2022). In contrast, non-thermal extraction techniques, such as HPP, offer an effective alternative that enables the preservation and enhancement of bioactive compound yields, such as ACNs, without compromising their integrity. Therefore, exploring HPP to recover bioactive compounds from MP is crucial.

Addressing these challenges and leveraging the potential of by-products aligns with SDG goals 3 and 12. SDG 3 focuses on maintaining health and well-being for all ages; therefore, this study will contribute by utilizing the bioactive compounds from MP, especially ACNs, in the development of functional foods or natural colorants for foods and beverages, pharmaceuticals, and the nutraceutical industry. In addition, the utilization of MP directly supports SDG 12 by valorizing MP instead of discarding it as waste, thereby reducing greenhouse gas emissions and promoting the efficient use of natural resources for developing value-added products. Focusing on that, this study aimed to evaluate the effect of extraction using maceration and HPP on ACNs, phenolics, flavonoids, antioxidants, PPO, and POD enzymes and the color quality of dried MP extracts.

## 2 Materials and methods

### 2.1 Chemicals

4,6-tripryridyl-*s*-triazine (TPTZ), Folin-Ciocalteu reagent, gallic acid, and 2,2-Diphenyl-2-picrylhydrazyl (DPPH) were obtained from Sigma-Aldrich (St. Louis, MO, USA). Catechol (99 %) was obtained from Acros Organics (New Jersey, USA). Guaiacol 99 % was purchased from Thermo Scientific (New Jersey, USA). Standard chemicals: cyanidin-3-*O*-sophoroside (C3S), cyanidin-3-*O*-glucoside (C3G), and pelargonidin-3-*O*-glucoside (P3G) were purchased from Systerm (Selangor, Malaysia). Other chemicals and solvents were of analytical grade and purchased from Merck (Darmstadt, Germany).

### 2.2 Sample preparation and processing

Fresh purple mangosteen (index 6) was purchased from a local market in Serdang, Selangor, Malaysia. Fresh mangosteen was cleaned under running water, and MP was separated from the flesh and cut into small pieces before blanching.

The MP (50 g) was placed in the steam drawer and steamed for 90 s. Steam blanching was performed using a steamer (SR-Y22FGJ, Panasonic Appliances Co., Ltd. India) with 3 L of distilled water preheated to 100 ± 1 °C. The steamed MP was immediately transferred to ice water to stop the blanching process. 600 g of steamed MP was freeze-dried in a freeze drier (Labconco, Kansas City, MO, USA) at −45 ± 1 °C for 36 h. For the preparation of dried MP powder, dried MP was ground for 2 min, passed through a 0.50 mm (35 mesh) sie*v*e, and kept in a freezer at −20 °C until further analysis. For the extraction process, 6 g of freeze-dried MP powder was mixed with 60 mL of 50 % ethanol (EtOH) (*v*/v) (1:10 *w*/*v*) in a 60 mL polyethylene terephthalate (PET) bottle.

#### 2.2.1 HPP extraction

The MP extract was subjected to a non-thermal extraction using a 55 L capacity HPP unit (Hyperbaric, Burgos, Spain) located at the Faculty of Food Science and Technology, Universiti Putra Malaysia. A preliminary study was conducted using different ratios of water to EtOH as solvents and different pressures (300–600 MPa) at fixed times of 3 min. The optimal pressure (500 MPa) and solvent concentration (50 % EtOH aqueous solution) at 20 ± 1 °C were selected. The extraction time was set at 2–10 min (2 min intervals), and water was used as the pressure-transmitting medium.

#### 2.2.2 Maceration

Based on a preliminary study, 3 h of maceration of dried MP at 20 ± 1 °C was selected out of 24 h, as determined using total monomeric ACNs content (TMAC) (Nawawi et al., 2023a). 60 mL of 50 % EtOH aqueous solution was added to 6 g of dried MP and left to macerate in the dark at 20 ± 1 °C for 3 h. The dried MP extract was then *v*acuum-filtered in a Buncher funnel *v*acuum and centrifuged at 1030 ×*g* for 15 min before analysis.

### 2.3 High-performance liquid chromatography (HPLC) analysis of ACNs

HPLC analysis followed Azman, Nor, Charalampopoulos, and Chatzifragkou (2022) with slight modifications. The concentration of ACNs in the extracts was measured at 30 °C using a Purospher STAR RP18 end-capped column (250 mm × 4.6 mm i.d., particle size of 5 μm, Merck, Darmstadt, Germany) in a Perkin Elmer Series 200 HPLC system (PerkinElmer Inc., Bridgeport Avenue Shelton, U.S.A), equipped with a Perkin Elmer Series 200 UV/Vis detector, Perkin Elmer Series 200 pump, Shimadzu CTO-10 A column oven with a manual injector and Perkin Elmer Series 200 vacuum degasser. The mobile phase contained 2 % (*v*/v) formic acid (Solvent A) and 100 % (v/v) methanol (Solvent B). The gradient elution system involved 15 % (B) at 0 min, 35 % (B) at 15 min, 60 % (B) at 30 min, and 80 % (B) at 40 min. The injection volume was maintained at 20 μL, and the flow rate was set at 1.0 mL/min. Analysis was performed for 45 min at a wavelength of 520 nm. Individual ACNs were quantified using external standard calibration curves with the following concentrations range: 0.01–0.1 mg/mL (ACNs). The determination coefficient (R^2^), the limit of detection (LOD), and limit of quantification (LOQ) were computed using Eq. 1:

(1) $$\mathrm{LOD}=\frac{3\mathrm{Sa}}{b}\;\mathrm{and}\;\mathrm{LOQ}=\frac{10\mathrm{Sa}}{b}\;$$

where *Sa* is the standard deviation of the response and *b* is the slope of the calibration curve.

### 2.4 Determination of total phenolic content (TPC)

The TPC was determined using the Folin-Ciocalteu method described by Azman, Charalampopoulos, and Chatzifragkou (2020) with minor modifications. The absorbance of the samples was measured at 765 nm using a spectrophotometer (Thermo BioMate 3, Waltham, MA, USA). Gallic acid (0–100 mg/L) was used as a standard for the calibration curve and expressed as milligrams of gallic acid equivalents per gram (mg GAE/g). Measurements were performed in triplicate, and the mean values were calculated.

### 2.5 Determination of total flavonoid content (TFC)

TFC was determined using an aluminum chloride colorimetric assay described by Yea, Nevara, Muhammad, Ghazali, and Karim (2019) with minor modifications. The absorbance of the samples was measured at 510 nm using a spectrophotometer and was expressed as milligrams of catechin equivalent per gram (mg CE/g) of the sample. The standard calibration curve used a catechin solution (0–300 μg/mL).

### 2.6 Determination of antioxidant activities

***DPPH radical scavenging activity**.* The capacity of the MP extract to scavenge the DPPH radical was evaluated according to Ezzat, Abetra, Noranizan, and Yusof (2020) and Zhou, Lin, Wei, and Tam (2011) with modifications. The absorbance of the samples was measured at 517 nm using a spectrophotometer. The results are expressed in μmol Trolox equivalent per gram (μmol TE/g) of the MP sample.

***Ferric-reducing antioxidant power (FRAP) assay.*** The FRAP was analyzed as described by Senevirathna, Ramli, Azman, Juhari, and Karim (2021) with minor modifications and observed spectrophotometrically at 593 nm. The results were derived from a standard curve of Trolox solution (0–2000 μM) and expressed in μmol of Trolox equivalent per gram of the sample (μmol TE/g).

### 2.7 Determination of color characteristics

A chroma meter (CR-410, Konica, Minolta, Japan) was used to measure the color of the MP extracts and reported as L*, a*, and b*. The total color difference (∆E), hue (h°), and chroma (C*) were calculated using Eqs. (2), (3), (4):

(2) $$\text{Total color difference}\;\left(∆E\right)={\left[{\left(L^{*}-L_{0}^{*}\right)}^{2}+\left(a^{*}-a_{0}^{*}\right)+{\left(b^{*}-b_{0}^{*}\right)}^{2}\right]}^{\frac{1}{2}}\;$$

(3) $$\mathrm{Hue}\;\left(h^{{}^{\circ}}\right)=\tan^{-1}\left(\frac{b^{*}}{a^{*}}\right)$$

(4) $$\text{Chroma}\;\left(C^{*}\right)=\sqrt{(a^{*2}+b^{*2}}\;$$

where L_o_^⁎^, a_o_^⁎^, and b_o_^⁎^ are the values of the control samples extracted at 1 min during maceration and HPP, respectively. All measurements were performed in triplicate and the results were averaged.

### 2.8 Determination of browning index (BI)

The BI assessment was modified by Lee, Seo, Rhee, and Kim (2016). The MP extract was absorbed at 420 nm using a spectrophotometer. All measurements were performed in triplicate and the results were averaged.

### 2.9 REA: Polyphenol oxidase (PPO) and peroxidase (POD)

***PPO assay*:** The REA of PPO was assayed according to Deylami et al. (2016) with slight modifications, and the absorbance was monitored for 5 min at 1-min intervals at 420 nm using a spectrophotometer.

***POD assay*:** The POD residual activity was assayed according to Deylami, Rahman, Tan, Bakar, and Olusegun (2014) with minor adjustments. The absorbance was monitored using a spectrophotometer for 5 min at 1-min intervals at 470 nm.

Triplicate data were obtained, and both PPO and POD activities are shown as REA in percentage (%) according to Eq. 5:

(5) $$\mathrm{REA}\;\left(\%\right)=\frac{A_{t}}{A_{0}}\times 100\%$$

where A_t_ and A_0_ are the specific enzyme activities in the MP extracts after extraction and the control, respectively.

### 2.10 Thermal degradation studies

The thermal degradation of ACNs was studied at 60, 70, and 80 °C. MP extracts (15 mL) were placed in a 30 mL amber bottle and immersed in a shaking water bath at 180 rpm. 1 mL of extracts were taken at 2 h intervals (0−12*h*), and the total ACNs were determined using the pH differential method described below by Nawawi et al. (2023a).

(6) $$C_{\text{ACNs}}=\frac{A\times \mathrm{MW}\times \mathrm{DF}\times 10^{3}}{\varepsilon \times 1}$$

where C_ACNs_ (mg/L) = total monomeric ACNs concentration (A_520nm_ – A_700nm_)_pH1.0_ – (A_520nm_ – A_700nm_)_pH4.5_; MW (C3G equivalents, mg/L); A (absorbance) = (molecular weight) = 449.2 g/mol for C3G; extinction coefficient in L/mol/cm for C3G; DF = dilution factor; l = path length in cm; ε = 26,900 M and 10^3^ = factor for conversion from g to mg.

The ACNs content (mg/g) was then calculated as follows:

(7) $$\text{ACNs content}\;\left(\frac{\mathrm{mg}}{g}\right)=\frac{C_{\text{ACNs}}\left(\frac{\mathrm{mg}}{L}\right)\times \text{extract}\;\left(L\right)}{\text{Sample powder}\;\left(g\right)}$$

The degradation study and temperature dependency of ACNs were determined according to Chisté, Lopes, and De Faria (2010). The zero-order kinetic reaction was fitted to the reaction and calculated using Eq. 8:

(8) $$C_{t}=C_{0}-kt$$

C_0_ is the initial concentration of ACNs, C_t_ is the corresponding concentration at time (t), *k* is the rate constant, and t is time (h). The half-life time (t_1/2_) from the zero-order was determined from Eq. 9:

(9) $$t_{\frac{1}{2}}=\frac{\left[C_{0}\right]}{2k}\;\left(\text{half}-\text{life from zero}-\text{order reaction}\right)$$

where *k* is the rate constant of zero-order reaction.

The temperature dependency of ACNs degradation was determined using the Arrhenius equation by calculating the activation energy (Ea) using Eq. 10. The temperature quotient (Q_10_) was calculated according to Eq. 11.

(10) $$k=k_{0}e^{-\mathrm{Ea}/\mathrm{RT}}$$

(11) $$Q_{10}={\left(\frac{k_{2}}{k_{1}}\right)}^{10/\left(T_{2}-T_{1}\right)}$$

where, k, k_1,_ and k_2_ are the degradation constants rate (h^−1^); k_0_ is the pre-exponential factor; Ea is the activation energy (kJ/mol); R is the general constant gases (8.314 J/mol·k); T is the absolute temperature in K; Q_10_ is the temperature quotient; T_2_ and T_1_ are the temperatures (°C).

### 2.11 Fourier-transform infrared spectroscopy (FT-IR) analysis

Dried MP extract was freeze-dried and tested by Fourier-transform infrared spectroscopy (FT-IR) using a Bruker IN*V*ENIO® FT-IR Spectrometer with Universal Attenuated Total Reflectance (UATR) technique. The spectra were read in the 400–4000 cm^−1^ spectral region, with a resolution of 4 cm^−1^ and 32 scans.

### 2.12 Morphological changes in MP

Scanning electron microscopy (SEM) analyses were conducted to observe changes in the morphological structure of the extracted dried MP. The samples were analyzed microscopically using a scanning electron microscope (JSM-IT 100, JEOL Ltd., Japan). The samples were adhered to the stub using clean double-sided tape and coated with gold particles before viewing at 5000 × magnification at 10 kV.

### 2.13 Statistical analysis

All statistical analyses were conducted using one-way and two-way analysis of variance (ANOVA). Tukey's multiple range tests were used, with a probability of *p* ≤ 0.05. Linear Pearson correlation was also applied to evaluate correlations between ACNs, TPC, TFC, residual PPO and POD enzyme activities, antioxidant activity, and color characteristics. Statistical and multivariate analyses (General Linear Model (GLM)) and the correlation coefficient using correlogram analysis were conducted using Minitab V.21 (Minitab Inc., State College, PA, USA). Principal Component Analysis (PCA) was analyzed using OriginPro 2024 (OriginLab, Northampton, MA, USA).

## 3 Results and discussion

### 3.1 Extraction yields and anthocyanins (ACNs)

Total ACNs during maceration and HPP ranged from 2.66 to 6.01 mg/g. As shown in Table 1, longer processing times during HPP extraction significantly (p ≤ 0.05) affected the ACNs, whereby HPP-10 min demonstrated the highest total ACNs. Maceration and HPP-2 min indicated the lowest total ACNs with no significant difference between samples. This result suggests the significant impact of HPP on enhancing the extraction yield compared to the conventional method.

**Table 1:** ACNs composition in MP after maceration and HPP extraction as measured using HPLC.

Extraction method	ACNs (mg/g)			
	C3S	C3G	P3G	Total
Maceration (3 h)	3.12 ± 0.06^Ad^	< LOD	nd	3.12 ± 0.06^f^
HPP-500 MPa/2 min	2.66 ± 0.02^Ae^	< LOD	nd	2.66 ± 0.02^f^
HPP-500 MPa/4 min	3.89 ± 0.10^Ac^	< LOD	nd	3.89 ± 0.10^e^
HPP-500 MPa/6 min	4.24 ± 0.01^Ac^	0.14 ± 0.00^Ba^	nd	4.38 ± 0.01^d^
HPP-500 MPa/8 min	4.82 ± 0.27^Ab^	0.18 ± 0.02^Ba^	nd	5.00 ± 0.28^c^
HPP-500 MPa/10 min	5.78 ± 0.06^Aa^	0.23 ± 0.02^Ba^	nd	6.01 ± 0.08^a^
LOD (mg/mL)	0.01	0.01	0.01	
LOQ (mg/mL)	0.03	0.02	0.02	

Values with the same letter ^a-f^ in each row are not significantly different (*p* ≤ 0.05). Values with the same letter ^A-F^ in each column are not significantly different (*p* ≥ 0.05). HPP, high pressure processing; C3S, cyanidin-3-*O*-sophoroside; C3G, cyanidin-3-*O*-glucoside; P3G, pelargonidin-3-*O*-glucoside; nd, not detected; LOD, limit of detection; LOQ, limit of quantification.

**Fig. 1:**
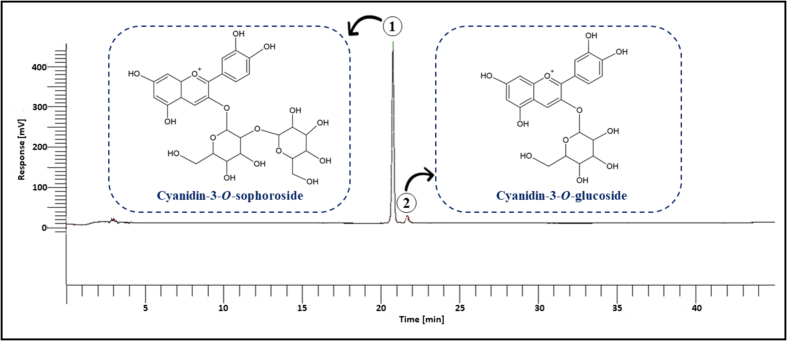
Typical ACNs detected in dried MP extract as quantified using HPLC at 520 nm. (1) - Cyanidin-3-*O*-sophoroside (C3S); (2) - Cyanidin-3-*O*-glucoside (C3G).

Maceration, which relies on the diffusion of solvents through plant cells, is time-consuming and often less effective than HPP. The improvement in extraction yield with HPP can be attributed to the high pressure exerted on the plant cells. This pressure eventually causes cell rupture and the release of membrane-bound compounds that are not accessible through the maceration process. To the best of our knowledge, the present study is the first report on extracting ACNs from MP by using HPP. C3S was the main ACNs found in the extract, followed by C3G (Fig. 1). However, P3G was not detected, similar to the findings of Nawawi et al. (2023a).

The increase in ACNs in HPP-10 min is related to cell rupture due to the applied pressure (da Silveira et al., 2019). These findings are consistent with de Jesus, Cristianini, Santos, and Júnior (2020), who reported an increase of 37 % in ACNs in acai pulp after being subjected to 600 MPa/3 min, corroborating the high pressure improved extractability of ACNs. At constant pressure, prolonged HPP time caused damage to the integrity of the cell walls of MP, enhancing the extraction of individual and total ACNs, as confirmed by the results at 6–10 min of HPP. Owing to the principle of uniformity of the pressure in HPP, the pressure-sensitive non-covalent bonds (hydrogen, ionic, and hydrophobic bonds) (Nawawi et al., 2023b; Nowacka et al., 2021) in the cell wall and membranes of MP were altered which assisted in the diffusion of the bound and bound-free ACNs from the inner matric plant cells to the solvent. In this case, the disrupted cells enhanced the penetration of polar solvents into the plant cells, thereby increasing the mass transfer of ACNs owing to their high solubility in the solvent. Unlike thermal extraction, HPP pressure does not affect the covalent bonds in the ACNs core structure, eventually protecting the sugar moieties of C3S and C3G from undergoing cleavage, hydrolysis, or oxidation, which triggered their degradation and formation of chalcone, an unstable form of ACNs. This suggests the effectiveness of HPP in preserving and enhancing the yield of heat-sensitive ACNs in MP.

Each extraction method was capable of extracting C3S from MP. In contrast, only maceration, HPP-2 min and HPP-4 min were below the detection limit for C3G. This indicates that the low extractability of maceration owing to the low mass transfer from the matrix cell of MP. Despite having the lowest yield among the samples, the total ACNs in maceration were still comparable to the ACNs observed by Nawawi et al. (2023a). In their findings, 2.11–2.20 mg/g was obtained after freeze-drying for 36 and 48 h, followed by extraction in a shaking water bath at 50 °C for 2 h. Azman et al. (2016) reported that C3G is one of the bound ACNs in dried blackcurrant, possibly similar to dried MP. Therefore, the high bound-free C3S content in dried MP may explain its extractability in maceration. The low concentration of C3G during HPP-2 min and HPP-4 min was due to the shorter extraction time, which was insufficient to extract C3G completely.

However, P3G was not detected in any of the extraction methods, possibly due to its low concentration in MP and the influence of the solvent. ACNs are sensitive to various factors, including light, enzymes, heat, and pH (Benvenutti, Zielinski, & Ferreira, 2022; Nawawi et al., 2023a). In this case, the less acidic solvent is probably the reason for the undetectable P3G peak in the extract. Azman et al. (2020) pointed out that acidic solvents enhanced the extraction of free ACNs from dried blackcurrant at 30 and 50 °C. Therefore, the solvent pH must be considered when extracting less concentrated or sensitive compounds, such as ACNs.

### 3.2 Total phenolic content (TPC)

**Fig. 2:**
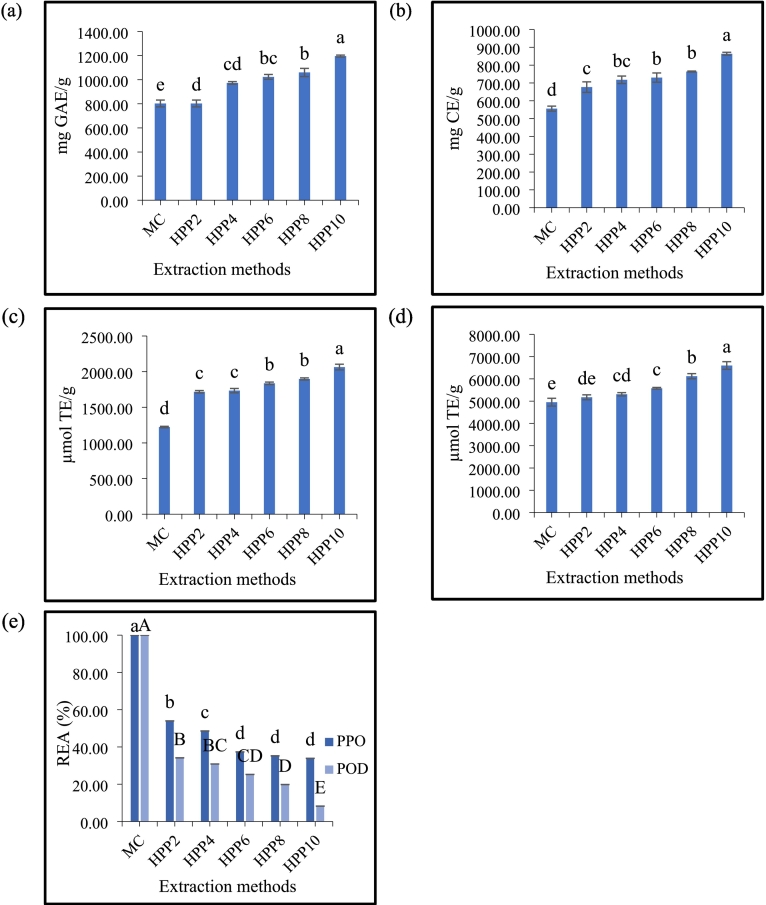
The effect of maceration (3 h/20 ± 1 °C) and HPP extraction (2–10 min/500 MPa/20 ± 1 °C) on (a) TPC, (b) TFC, (c) scavenging activity, (d) reducing power and (e) PPO and POD enzymes of dried MP extracts. All measurements were made in triplicate (*n* = 3), and results were averaged. Values with the same letter ^a-e^ or ^A-E^ are not significantly different (*p* ≥ 0.05). MC: maceration; HPP: high-pressure processing. HPP followed by number indicates time (min).

As shown in Fig. 2(a)**,** the TPC ranged from 802.84 to 1196.35 mg GAE/g, with maceration reported as the lowest (*p* ≤ 0.05). A significant increase in TPC was observed over time in the HPP samples, whereby HPP-10 min exhibited the highest phenolic content. These results are in line with those of da Silveira et al. (2019), who studied the effects of HPP (400, 450, 500, and 600 MPa/5 min) and thermal pasteurization (85 °C/1 min) on acai juice. They observed the highest phenolic content in HPP at 500 MPa/5 min and the lowest in the thermally pasteurized sample, suggesting that a combination of optimized pressure and time improved phenolic content.

**Fig. 3:**
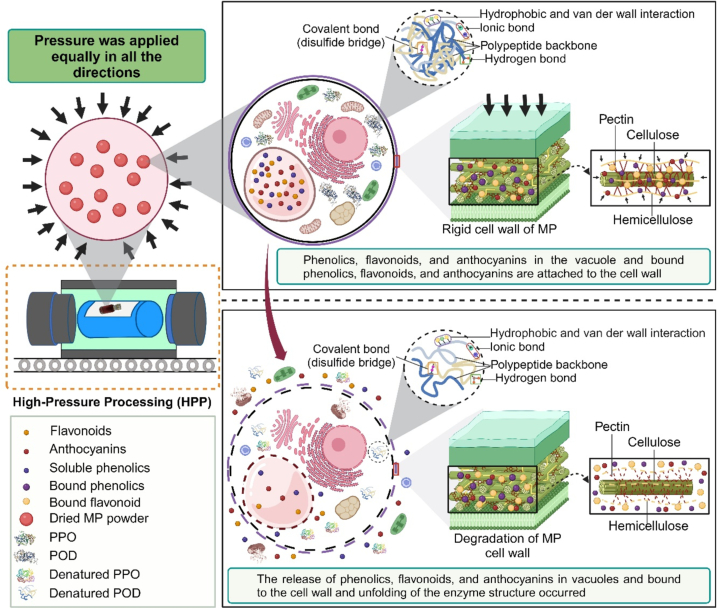
Mechanism of HPP on disruption of plant cells during extraction of dried MP powder.

The lowest phenolic content observed after maceration was likely due to lesser disruption of the MP cell wall. The maceration samples were unaffected by external forces, such as pressure, which occurred in the HPP samples. HPP increases the pressure difference between the inside and outside of the cell (Liu et al., 2016), resulting in damage to the cell membrane and cell rupture, thereby releasing bioactive compounds (Fig. 3). Therefore, the release of phenolic compounds from the matrix cells during maceration extraction was presumably influenced by the particle size of the freeze-dried MP powder and polarity of the solvent. The small size of the MP powder increased the surface area. It assisted in the permeability and diffusion of the solvent into the cell, which resulted in the high extractability of bound-free phenolics in the matrix cell of MP. Meanwhile, the polarity of the solvent improved the extraction of polar compounds, such as ACNs.

Compared to the HPP-2 min, the phenolic content increased by 22.96 % after 10 min. These results contrast the findings of Zhang et al. (2021), who revealed that the TPC value of freeze-dried red raspberries decreased to 20.60 % after HPP at 600 MPa/10 min. These findings could be related to the higher pressure applied to extract red raspberries than that used in the present study, which may not favor the bioactive compounds in red raspberries. Notably, in the current study, the phenolic content remained stable within 2–4 min and 6–8 min of HPP, except for a significant increase at 10 min, suggesting the release of bound-free phenolics during the first 4 min. Therefore, prolonged extraction time caused alterations in dried MP's cell wall and membrane structure, successfully releasing the bound-membrane phenolic, as indicated by high phenolic in HPP for 10 min than in the maceration. The disruption of non-covalent bonds in the cell wall components caused a weakening in the binding of bound phenolics in MP cells. Besides, HPP induces the alteration and dissociation of the hydrogen, ionic and ester bonds in pectin, which commonly binds to compounds such as flavonoids (Hilz et al., 2006; Nowacka et al., 2021). Thereby, HPP can be an effective technique to enhance the extraction of bound or bound-free phenolics from dried MP within a short duration.

### 3.3 Total flavonoid content (TFC)

Fig. 2(b) shows the changes in the TFC during maceration and HPP. The TFC ranged from 555.85 to 863.73 mg CE/g. Maceration showed the lowest values. Meanwhile, in the HPP extraction samples, no significant differences (*p* ≥ 0.05) were observed between 2 and 4 min and from 4 to 8 min, except at 10 min. Overall, HPP-10 min exhibited the highest TFC values (1.55-fold) higher than maceration, indicating that flavonoids increased in a time-dependent manner rather than the pressure level.

These findings align with those of Zhang et al. (2021), where red raspberries treated with HPP at 600 MPa/10 min/25 °C showed an increase of 52.67 % in flavonoid content. In addition, Tenuta, Artoni, Fava, Bignami, and Licciardello (2023) observed an increase of 32.60 % in TFC in sour cherries after being subjected to 600 MPa/3 min/4 °C. The hypotheses of high pressure and longer processing duration in HPP induced the extractability of total flavonoids in the MP extract, supported by Rios-Corripio, Welti-Chanes, Rodríguez-Martínez, and Guerrero-Beltrán (2024). They found that high pressure (>200 MPa) and extended processing resulted in high concentrations of flavonoids in black cherries. The localization of flavonoids in MP matrix cells influences the flavonoid content. Despite the lowest TFC value, the maceration sample was comparable to the TFC value (621.0 mg CE/g) obtained by Nawawi et al. (2023a). Therefore, the release of flavonoids during maceration is primarily caused by bound-free flavonoids dissolved in the solvent.

The increased flavonoid content during HPP suggests that constant pressure and prolonged processing duration modify internal and external structures (cell walls, membrane alterations, vacuoles, organelles) and decompartmentalize flavonoids. Once the cell wall and cell membrane are altered, isostatic pressure causes the release of bound flavonoids, and the rupture of the vacuole leads to an increase in soluble flavonoids (Rux, Gelewsky, Schlüter, & Herppich, 2019). Thus, soluble and insoluble flavonoids accumulate in the extracts.

### 3.4 Effects of HPP on antioxidant activities

Maceration resulted in significantly the lowest scavenging activity and reducing power (*p* ≤ 0.05), respectively (Fig. 2(c) and Fig. 2(d)). This phenomenon was aligned with the changes in the content of ACNs, TPC, and TFC, as discussed previously. Meanwhile, HPP extraction showed no significant differences in scavenging activity within 2–4 min and 6–8 min (*p* ≥ 0.05). Besides, prolonged HPP extraction positively affects the antioxidant activities of MP extract. The highest antioxidant activity was observed for HPP at 10 min. The increase in low molecular weight molecules (volatile compounds, pigments, and vitamins) could be the reason for the high levels of antioxidants in HPP at 10 min (Nawawi et al., 2023b).

The highest increase in scavenging activity and reducing power in the MP extract sample after being subjected to 500 MPa/10 min was due to the longer duration applied to the cell wall and membrane of the MP, resulting in the breakdown of pectin, hemicellulose, and cellulose (Fig. 3). The ester bonds of pectin are hydrolyzed during HPP, causing the alteration and breaking of the methyl ester linkages in MP. Therefore, the highly methyl-esterified form of pectin weakens and affects cell structure. In addition, cellulose, which is made of glucose and is linked with β-1,4-glycosidic bonds, is not directly affected by HPP; however, this cellulose interacts with other cell wall components via non-covalent bonds, which can be affected by HPP (Hilz et al., 2006). Thereby, the disruption in this interaction will also affect cellulose integrity. This phenomenon also occurs in hemicellulose due to their non-covalent interaction with other cell wall components and affects the overall cell wall and membrane structures, leading to disruption of cell integrity. Therefore, increases cell permeability, mass transfer, and the release of matrix-bound phenolic compounds in MP (Nawawi et al., 2023b; Yuan et al., 2022).

Both scavenging activity and reducing power of MP extract showed improvement as increased extraction time in HPP, suggesting the release of compounds such as ACNs monomers, xanthones, and tannins, possibly caused synergistic action, such as intermolecular copigmentation and self-association between other compounds (phenolics, flavonoids, amino acids, and carbohydrates) which eventually contributed to the antioxidant activity (Liu et al., 2024). Furthermore, the ACNs, TPC, and TFC also contributed to high antioxidant activities, as indicated by the strong correlation between reducing power (ACNs: *r* = 0.922, TPC: *r* = 0.914, TFC: *r* = 0.900) and scavenging activity (ACNs: *r* = 0.759, TPC: *r* = 0.940, TFC: *r* = 0.947) at *p* ≤ 0.05. Thus, these findings suggest that prolonged HPP at 500 MPa enhanced the extraction of bioactive compounds in MP, resulting in greater antioxidant activities.

### 3.5 Polyphenol oxidase (PPO) and peroxidase (POD) enzyme activities

PPO and POD are endogenous enzymes related to browning in MP (Deylami, Rahman, Tan, Bakar and Olusegun, 2014, Deylami, Rahman, Tan, Bakar and Olusegun, 2016; Nawawi et al., 2023a). High levels of PPO and POD enzymes are undesirable because they accelerate the deterioration of perishable products, triggering the formation of *o*-quinone (melanin). Significantly higher REA values for PPO and POD were observed during maceration (p ≤ 0.05) (Fig. 2(e)).

A high REA in maceration was expected because this method does not apply pressure or heat to inactivate enzyme activities. PPO and POD were gradually reduced (p ≤ 0.05) in HPP extraction over time, where the REA of PPO and POD ranged from 54.00 % - 33.90 % and 34.16 % - 8.27 %, respectively. The inactivation of PPO exceeded 50 % after 4 min of HPP; however, consistent results were observed at 6–10 min (*p* ≥ 0.05). These findings suggest that a longer duration or higher pressure is required to increase the inactivation rate of PPO. Despite the inability to inactivate POD completely, POD showed better inactivation than PPO in all HPP samples. The more significant inactivation of POD after HPP treatment suggests that it is more pressure-sensitive than PPO, which aligns with the findings of Terefe, Yang, Knoerzer, Buckow, and Versteeg (2010).

Inactivation of PPO and POD in HPP due to high pressure and longer duration was exerted on the cell membrane, which affected the conformational structures of the enzymes. Weak bonds, such as ionic, hydrophobic, and hydrogen bonds, are disrupted, causing irreversible denaturation and unfolding of labile enzymes in MP extracts (Fig. 3) (Li & Padilla-Zakour, 2021; Szczepańska, Barba, Skąpska, & Marszałek, 2020). Various studies have documented the inactivation of PPO and POD under HPP treatment; however, these findings remain inconclusive, whereas others have reported decreased activity (Li & Padilla-Zakour, 2021; Szczepańska et al., 2020) and increased activity (Liu et al., 2016; Wibowo et al., 2019; Zhang et al., 2021).

Zhang et al. (2021) reported increased PPO and POD activities in red raspberries after extraction by HPP at 600 MPa/10 min. The activation of PPO activity was similar to the findings of Wibowo et al. (2019), where more than 100 % PPO activity was activated after 400 MPa/3 min and 600 MPa/3 min, respectively. In contrast, Tsikrika, O'Brien, and Rai (2019) pointed out that 59 %, 47 %, 51 %, and 69 % of PPO was inactivated in potatoes after 600 MPa/3 min of pressure was applied to different varieties of potatoes, which is in agreement with Li and Padilla-Zakour (2021) where 75.20 % and 80.70 % of PPO and POD remained in grapes was observed after subjected to 600 MPa/3 min. Different fruits (species and variety) vary in the type, isoforms, concentration, and pressure sensitivity of enzymes, leading to different pressure inactivation kinetics, resulting in partial or complete inactivation of enzymes during HPP processing (Kaur et al., 2020; Tsikrika et al., 2019). This explains the different inactivation rates of PPO and POD observed in HPP samples. The negative correlation (*p* ≤ 0.05) between ACNs, PPO (*r* = −0.663), and POD (*r* = −0.621,) confirmed the inactivation of PPO and POD, leading to an increase in ACNs. PPO and POD are common enzymes found in MP that promote the oxidation of *o*-diphenols to produce *o*-quinones and the *o*-hydroxylation of monophenols to produce *o*-diphenols (Deylami, Rahman, Tan, Bakar and Olusegun, 2014, Deylami, Rahman, Tan, Bakar and Olusegun, 2016; Ijod et al., 2022; Liu et al., 2016). This reaction negatively affects the stability of the ACNs. Therefore, inactivating PPO and POD can slow the reaction and increase the stability of the ACNs.

### 3.6 Color characteristics

Color reflects the quality of the products, and different processing methods influence the color quality of the targeted products. Table 2 shows the impact of extraction on the color of the MP extracts. The color changes reported in terms of lightness (L*), redness (a*), yellowness (b*), total color difference (ΔE), chroma (C*), and hue angle (h°) were compared between the color of the extracts and the controls (1 min extraction) and BI at 420 nm. Significantly higher L* and BI values were observed during maceration (*p* ≤ 0.05). Meanwhile, the lowest a* and C* values were observed in maceration, corresponding to high b* and h° values, respectively. There was a strong correlation (*p* ≤ 0.05) between the BI and PPO (*r* = 0.962) and POD (r = 0.962).

**Table 2:** Color characteristics, browning index (BI), and appearance of freeze-dried MP extracts obtained after extraction in maceration and HPP at 500 MPa.

Extraction methods/color properties	Maceration	HPP 2 min	HPP 4 min	HPP 6 min	HPP 8 min	HPP 10 min
L*	46.78 ± 0.20^a^	28.74 ± 0.21^e^	32.78 ± 0.18^d^	33.57 ± 0.35^c^	34.85 ± 0.23^b^	27.22 ± 0.19^f^
a*	22.36 ± 0.16^e^	30.84 ± 0.07^c^	31.72 ± 0.60^bc^	32.34 ± 0.47^b^	32.01 ± 0.21^b^	33.33 ± 0.20^a^
b*	29.93 ± 0.09^a^	10.81 ± 0.06^f^	15.16 ± 0.42^d^	15.88 ± 0.17^cd^	16.10 ± 0.21^c^	12.52 ± 0.62^e^
C*	25.49 ± 0.27^d^	32.68 ± 0.04^b^	35.15 ± 0.72^a^	36.03 ± 0.46^a^	35.83 ± 0.28^a^	35.84 ± 0.28^a^
**h°**	53.24 ± 0.14^a^	19.32 ± 0.14^f^	25.54 ± 0.21^d^	26.16 ± 0.27^cd^	26.70 ± 0.16^c^	20.45 ± 0.95^e^
**ΔE**	73.04 ± 2.06^b^	9.83 ± 0.52^d^	1.50 ± 0.72^f^	4.01 ± 1.06^ef^	7.03 ± 1.20^de^	14.50 ± 1.60^c^
**BI**	1.36 ± 0.05^a^	1.08 ± 0.02^b^	1.04 ± 0.03^b^	0.95 ± 0.03^c^	0.91 ± 0.02^c^	0.82 ± 0.01^d^
**The appearance of MP extracts and freeze-dried MP extracts**						

Each value represents the mean ± SD (*n* = 3). Values with the same letter ^a-f^ in each row are not significantly different (*p* ≥ 0.05). HPP: High pressure processing.

HPP has been reported to preserve color and has minimal impact on covalent bonds or low molecular weight compounds (Nawawi et al., 2023b; Wibowo et al., 2019). Compared to maceration, all HPP samples exhibited low L* values, with the lowest values observed in HPP at 10 min (p ≤ 0.05), corresponding to high a* and C*, and the lowest in b*. High a* and C* in HPP, especially at 10 min, were attributed to the ACNs as indicated by the positive correlation between ACNs and a* (*r* = 0.594) and C* (*r* = 0.623) at *p* ≤ 0.05. HPP samples had lower b* values, indicating less yellowness of the extract than maceration. The high yellowness observed in maceration was due to PPO and POD activity, as indicated by a strong positive correlation (*r* > 0.851, p ≤ 0.05) between b*, PPO, and POD.

A significantly higher reduction in BI was observed in HPP samples over time, suggesting the inactivation of PPO and POD enzymes and inhibition of the Maillard reaction, resulting in better color quality during HPP. In addition, a higher ΔE was observed in maceration than in HPP samples, indicating that HPP enhanced and preserved the color quality of MP extracts.

### 3.7 Thermal degradation of ACNs

The kinetic degradation of ACNs in both extraction methods followed a zero-order reaction with a regression coefficient (R^2^) ranging from 0.895 to 0.984 (Table 3). The degradation of ACNs in the maceration and HPP-10 min samples exhibited similar trends over time, except at 60 °C. At 60 °C, a lower rate of degradation of ACNs in macerated (0.049 h^−1^) and HPP (0.067 h^−1^) was recorded. However, the half-life (t_1/2_) of ACNs in HPP-10 min was significantly longer than t_1/2_ in maceration (p ≤ 0.05). These possibly due to the high concentration of bioactive compounds, such as phenolic and flavonoid compounds which promote synergistic effects that led to ACNs stability in HPP-10 min.

**Table 3:** Effect of maceration and HPP extraction on the estimated half-life time values (t_1/2_, h) of ACNs from dried MP extracts.

Temperature (°C)	Maceration					HPP (500 MPa, 10 min)				
	R^2^	k	t_1/2_ (h)	Q_10_	Ea	R^2^	k	t_1/2_ (h)	Q_10_	Ea
60	0.947	0.049	17.75 ± 1.37^Aa^	1.592 (60 °C - 70 °C)	45.00	0.895	0.067	20.31 ± 0.40^Ba^	1.687 (60 °C - 70 °C)	37.21
70	0.885	0.078	12.19 ± 0.44^Ab^			0.966	0.113	12.72 ± 0.58^Ab^		
80	0.984	0.123	7.98 ± 0.26^Ac^	1.577 (70 °C - 80 °C)		0.979	0.143	8.67 ± 0.69^Ac^	1.265 (70 °C - 80 °C)	

Values with the same letter ^a-c^ in each row are not significantly different (p ≥ 0.05). Values with the same letter ^A-C^ in each column are not significantly different (p ≥ 0.05). R^2^, coefficient determination; k, rate constant (h^−1^); t_1/2_, half-life (h); Q_10_, temperature quotient; Ea, activation energy (Kj/mol).

At 70 °C, approximately 52.71 % and 50.70 % of ACNs remained in the HPP sample (t_1/2_ = 12.72 h) and maceration (t_1/2_ = 12.19 h) after 12 h, respectively. At 80 °C, the degradation of ACNs exceeded 70 % in HPP-10 min and maceration. The t_1/2_ of HPP-10 min (8.67 h) and maceration (7.98 h) suggest that temperature and time are among the crucial factors that must be considered to preserve ACNs. Over time, an increasing ACNs degradation rate was observed for HPP-10 min and maceration. The t_1/2_ of ACNs becomes shorter as the temperature increases, which is expected owing to the heat-sensitive properties of ACNs. C3S and C3G in MP are non-acylated ACNs that do not possess high stability compared to acylated ACNs (Cai et al., 2022). This explains the significant degradation rate of ACNs. This could be linked to the breakdown of the glycoside linkages of C3S and C3G, which leads to the formation of aglycones. As heat is applied to the ACNs, several processes promote their degradation. The cleavage and hydrolysis processes in ACNs cause an opening in their core ring, eventually affecting the stability of individual ACNs in the extracts. Modesto, Junior, Pereira, Chisté, & Da Silva Pena, (2023) studied the thermal degradation of ACNs from Brazilian berries, and they observed that half of the ACNs were degraded after 10 h at 60 °C and 1.9 h at 80 °C. This shows that ACNs in the MP extract are more stable than ACNs from Brazilian berries.

The activation energy (Ea) was calculated to determine the energy required to initiate the reaction and observe the sensitivity of the ACNs to temperature variations. According to Table 3, the Ea of ACNs was 45.00 kJ/mol and 37.21 kJ/mol in maceration and HPP, respectively. These findings were 26.56 % and 39.52 % lower than the Ea of ACNs in MP after maceration extraction for 14 h, as reported by Chisté et al. (2010), suggesting that the concentration and types of ACNs significantly affect their stability to heat.

The temperature coefficient (Q_10_) indicates the sensitivity of the ACNs to temperature fluctuations. Similar Q_10_ values were observed at 60–70 °C and 70–80 °C in the ACNs after maceration. In contrast, ACNs extracted in HPP were significantly more susceptible to temperature changes of 60–70 °C, as indicated by the high Q_10_ compared with Q_10_ from 70 to 80 °C in HPP-10 min. A lower Q_10_ of ACNs was observed between 70 and 80 °C in the HPP sample. This suggests that the low Q_10_ observed between 70 and 80 °C might indicate that after a thermal threshold is reached, the ACNs either stabilize or the degradation rate decreases, possibly due to ACNs' interaction with other compounds, suggesting intermolecular or self-association to occur. This mechanism improves the structure of the ACNs by protecting their central rings, thereby increasing their stability. This behavior was also observed by Chisté et al. (2010), where storage in a low-temperature range of 5–35 °C caused a higher Q_10_ than storage at 28–40 °C and 40–50 °C. Therefore, it can be concluded that ACNs in the MP are more sensitive to temperature changes at higher temperatures than at lower temperatures. HPP offers better stability for ACNs at 60 °C than maceration; however, significant degradation of ACNs was observed when the temperature increased above 70 °C. Therefore, both the heat and extraction time should be carefully considered to preserve ACNs.

### 3.8 Fourier-transform infrared spectroscopy (FT-IR)

**Fig. 4:**
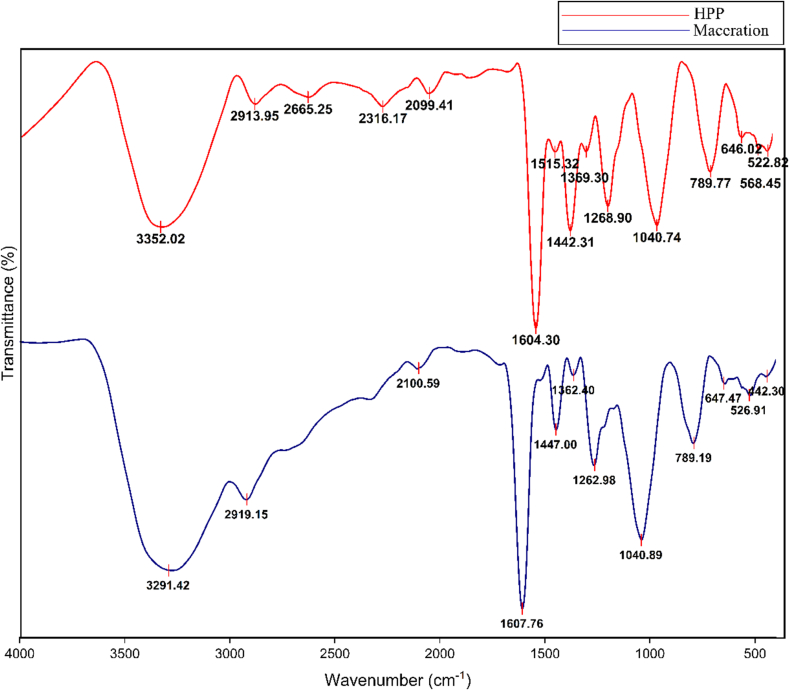
FT-IR of freeze-dried MP extract after extraction in maceration and HPP (500 MPa/10 min).

The results show broad bands at 3352.02 and 3291.42 cm^−1^ in maceration and HPP-10 min, indicating the stretching vibration of hydroxyl groups (OH) **(**Fig. 4**)**. Peaks at 2913.95 and 2919.15 cm^−1^ are the asymmetric stretching vibrations of C—H bonds of methylene groups (CH_2_). Notably, the weak peak of 2665.25 cm^−1^ only appeared in the HPP sample and corresponded to the N—H bond. In the context of MP, the N—H bond could be linked to protein fragments or other nitrogen-containing compounds that might have been released or exposed due to the HPP. This peak suggests that HPP could cause structural changes in the MP, leading to the exposure or formation of amine groups that were not as accessible or present in detectable quantities in the untreated or macerated samples. This could result from protein denaturation or the breakdown of other nitrogenous compounds during the HPP treatment. Additionally, peaks at 2316.17, 2099.41, and 2100.59 cm^−1^ suggest the presence of C≡N and C≡C (2000–2400 cm^−1^) in both maceration and HPP-10 min.

From the FT-IR spectra, maceration and HPP samples contained polar natural pigments as indicated by the carboxylic acid or alcohol groups detected, suggesting the influence of the polarity of the solvent. The sharp peaks at 1604.30 and 1607.76 cm^−1^ originated from the stretching vibration of the aromatic rings A and B of the ACNs; meanwhile, peaks around 1515.32, 1447.00, 1442.31, 1262.98, and 1268.90 cm^−1^ (1500–2000 cm^−1^) were originated from C-O-C aromatic rings of flavonoids (Bhushan et al., 2023; Tan et al., 2021). The wavenumber bands at 1040.74 and 1040.89 cm^−1^ indicate the stretching vibration of C-O-C from the glycosidic bond. The peaks ranged from 600 to 1000 cm^−1^, indicating the aromatic rings (Bhushan et al., 2023). The presence of these functional groups can be attributed to the chemical composition of the samples, which may influence the different extraction methods and the polarity of the solvent used.

### 3.9 Morphology of dried MP

**Fig. 5:**
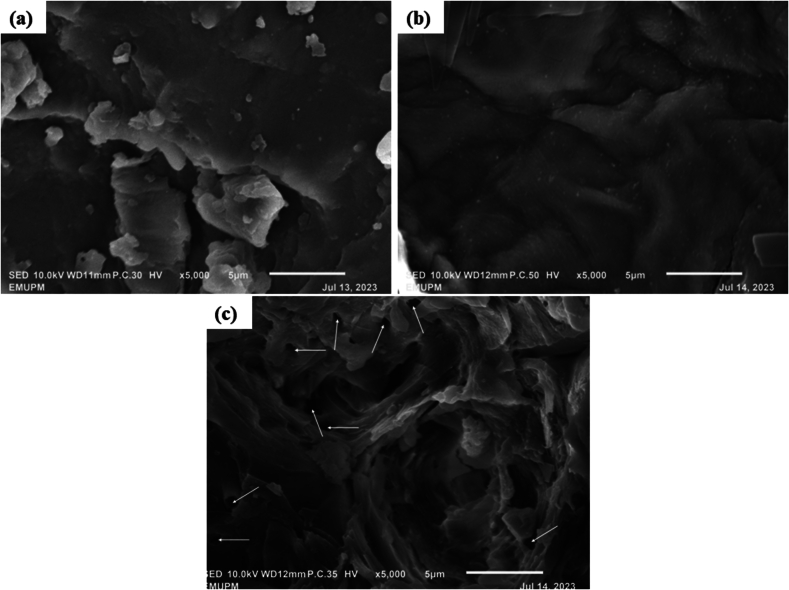
SEM images of dried MP powder processed by different conditions: (a) dried MP powder before extraction, (b) maceration, (c) HPP (500 MPa/10 min) at 5000 × magnification at 10 kV.

The effects of different extractions on the morphology of the dried MP powder before and after extraction were examined by SEM. The dried MP powder in Fig. 5(a) shows an intact structure, with flakes of particles on the surface, and no pores were observed. As shown in Fig. 5(b), maceration extraction did not have a detrimental impact on the cell structure, as a smooth and intact surface was observed, which was almost similar to the structure of dried MP.

Fig. 5(c) shows the structure of the dried MP powder of HPP-10 min at 500 MPa. Applying high pressure and longer extraction durations caused a more detrimental effect on the cell walls and membranes of the plants, as evidenced by the formation of pores. After HPP, the irregular pores in the dried MP cell walls and membranes suggest that the applied pressure caused different pressures inside and outside the cells, leading to damage and high porosity in the epidermal cells. The high porosity of dried MP during HPP extraction explains the high permeability and mass transfer of ACNs, phenolics, flavonoids, and antioxidants, thus assisting the high yield of bioactive compounds in the current study.

### 3.10 PCA and correlogram analysis

**Fig. 6:**
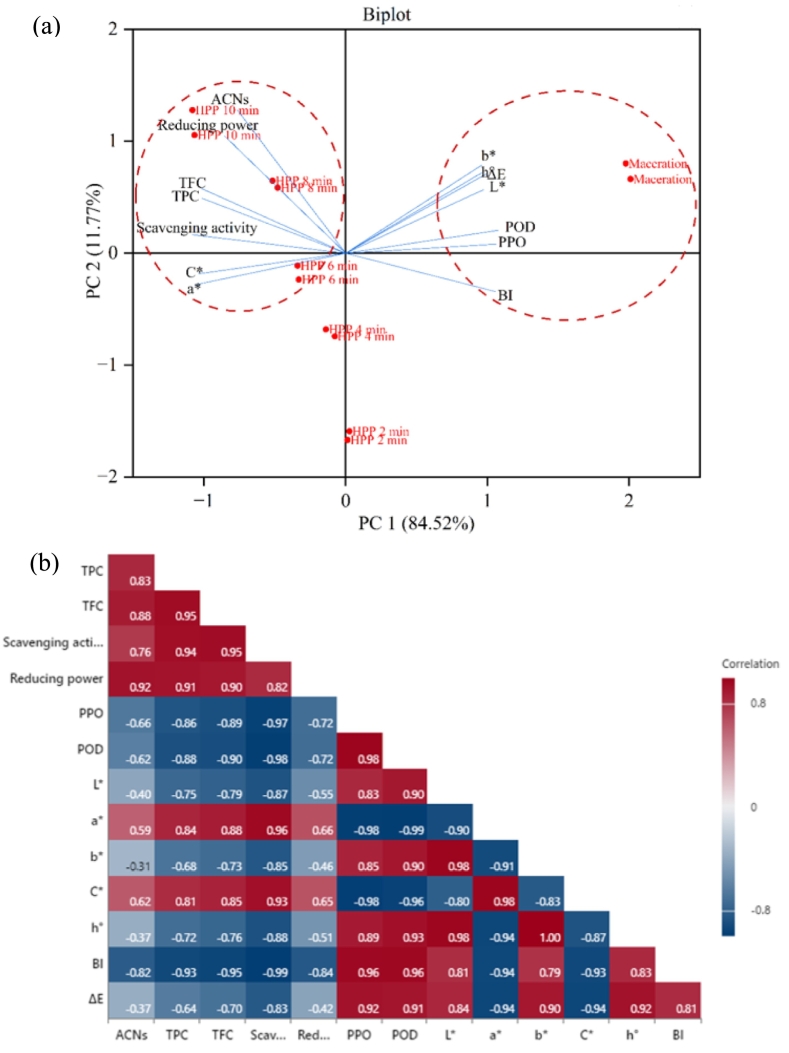
(a) Biplot PCA and (b) correlogram analysis of the effect of maceration and HPP on the physicochemical of dried MP extracts.

PCA was used to evaluate the effects of different extraction methods on ACNs, TPC, TFC, antioxidant activities, REA, and color properties (L*, a*, b*, h°, C*, and ΔE) and correlogram analysis were used to determine the relationship between variables using the Pearson correlation coefficient at *p* ≤ 0.05 **(**Fig. 6**)**. The variables and observations were assigned to the first two principal components (PC), which accounted for 96.29 % of the overall variation.

As depicted in Fig. 6(a), the first (PC 1) and second (PC 2) explained 84.52 % and 11.77 % of the total variation for the dried MP extracts, respectively. The positive scores on PC 1 corresponded with low color quality (L*, b*, h°, and ΔE) and high BI, PPO, and POD enzyme in the maceration sample, suggesting that enzymatic activity negatively affecting the color quality of the MP extracts. Meanwhile, the positive score in PC 2 was associated with high ACNs, antioxidant activity, TPC, TFC, and color quality (a* and C*) in the HPP samples, especially in HPP at 8- and 10-min. These results showed that the ACNs and other bioactive compounds gradually increase with the prolonged extraction in HPP, eventually improving the purity and redness of MP extracts. Therefore, HPP-10 min was the most effective method for enhancing the ACNs, bioactive compounds and color quality in dried MP extracts.

## 4 Conclusion

The extraction of ACNs from MP was the critical focus of this study. The results demonstrated that this emerging approach, particularly HPP (500 MPa/10 min), efficiently increased ACNs yield, phenolics, flavonoids, antioxidants, and color quality, with significant inactivation of PPO and POD enzymes compared to maceration. Notably, the presence of additional peak wavelengths in the HPP-10 min, which were absent during the maceration, emphasizes the superior efficacy of HPP as an extraction method. SEM further revealed the formation of pores in the HPP-10 min, suggesting that HPP is more effective than maceration in disrupting the cell structures. These results highlight the efficacy of HPP as an alternative to conventional extraction. Overall, the effectiveness of HPP in extracting bioactive compounds lies in its ability to physically disrupt cell structures while maintaining the integrity of heat-sensitive compounds, leading to higher extraction yields and better preservation of the bioactive properties of the extracted compounds. Furthermore, the results highlight the potential for valorizing freeze-dried MP as a natural colorant source in the food and pharmaceutical industry.

## CRediT authorship contribution statement

**Giroon Ijod:** Writing – original draft, Validation, Software, Methodology, Investigation, Formal analysis, Data curation. **Nur Izzati Mohamed Nawawi:** Writing – original draft, Methodology, Formal analysis, Data curation. **Rabiha Sulaiman:** Supervision, Methodology, Data curation, Conceptualization. **Mohammad Rashedi Ismail-Fitry:** Validation, Supervision, Resources, Data curation, Conceptualization. **Noranizan Mohd Adzahan:** Validation, Supervision, Formal analysis, Data curation, Conceptualization. **Farooq Anwar:** Writing – original draft, Validation, Supervision, Data curation, Conceptualization. **Ezzat Mohamad Azman:** Writing – review & editing, Validation, Supervision, Resources, Project administration, Funding acquisition, Conceptualization.

## Declaration of competing interest

The authors declare that they have no known competing financial interests or personal relationships that could have appeared to influence the work reported in this paper.

## Data Availability

Data will be made available on request.
